# Hunter Syndrome Diagnosed by Otorhinolaryngologist

**DOI:** 10.1155/2018/4252696

**Published:** 2018-05-13

**Authors:** Ayako Hashimoto, Tadayuki Kumagai, Hiroyuki Mineta

**Affiliations:** ^1^Department of Otorhinolaryngology, Shizuoka Children's Hospital, Shizuoka, Japan; ^2^Department of Pediatrics, Fujieda City General Hospital, Fujieda, Japan; ^3^Department of Otorhinolaryngology, Head and Neck Surgery, Hamamatsu University School of Medicine, Hamamatsu, Japan

## Abstract

Hunter syndrome is a lysosomal disease characterized by deficiency of the lysosomal enzyme iduronate-2-sulfatase (I2S). It has an estimated incidence of approximately 1 in 1,62,000 live male births. We report a case of Hunter syndrome diagnosed by an otorhinolaryngologist. To our knowledge, this is the first study diagnosed by an otorhinolaryngologist despite the fact that otorhinolaryngological symptoms manifest at a young age in this disease. The patient was a 4-year-old boy. He underwent adenotonsillectomy. Intubation was difficult, and he had some symptoms which are reasonable as a mucopolysaccharidosis. The otorhinolaryngologist should play an integral role in the multidisciplinary approach to the diagnosis and management of many children with MPS (mucopolysaccharidoses) disorders.

## 1. Introduction

Hunter syndrome (mucopolysaccharidosis type II (MPS II)) is a lysosomal disease characterized by deficiency of the lysosomal enzyme iduronate-2-sulfatase (I2S). It has an estimated incidence of approximately 1 in 1,62,000 live male births [1] and represents the major type of MPS disorder in East Asian countries. Patients with Hunter syndrome usually appear normal at birth, with clinical signs and symptoms manifesting between 2 and 4 years of age [2]. Clinical manifestations include severe airway obstruction, skeletal deformities, cardiomyopathy, mixed hearing loss, and, in most patients, neurological decline. Most patients exhibit ear, throat, and airway problems and, accordingly, visit ear, nose, and throat (ENT) clinics at a young age [3]. We report a case of Hunter syndrome, which, to our knowledge, is the first to be diagnosed by an otorhinolaryngologist despite the fact that otorhinolaryngological symptoms manifest at a young age in this disease.

## 2. Case Presentation

The patient was a 4-year-old boy, born at 38 weeks and 3 days of gestation, with a birth weight of 2110 g. He was examined by a neurologist for an arachnoid cyst and intellectual disability. He had a medical history of hospitalization for Kawasaki disease at 2 years of age and underwent operations for inguinal hernia at 10 months and 3 years of age. The patient was referred by an ENT physician from another general hospital, for airway obstruction and hearing loss. An informed consent from parents was obtained.

**Figure 1:**
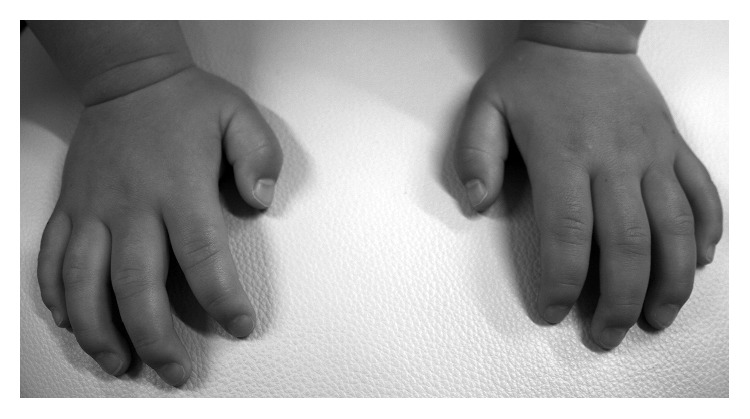
Hand X-ray imaging the clawhand deformity.

**Figure 2:**
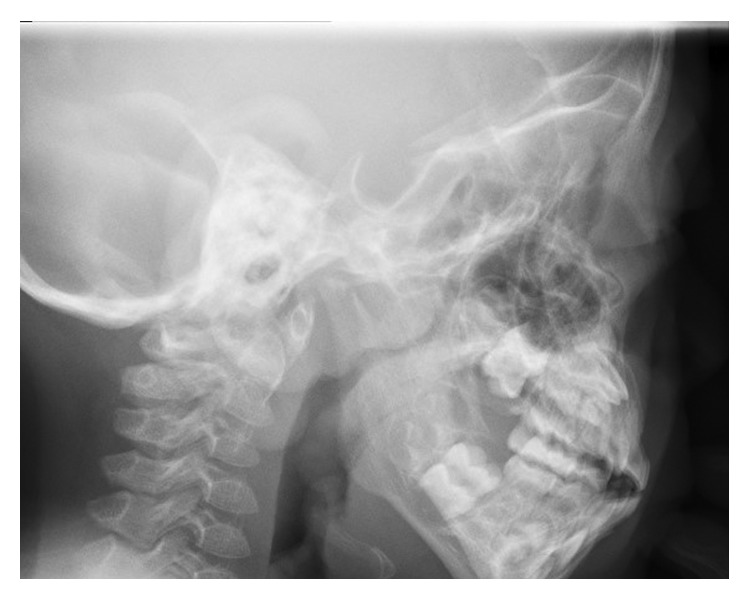
Lateral neck X-ray. Obstruction of the nasopharyngeal airway due to adenoid hypertrophy is apparent.

**Figure 3:**
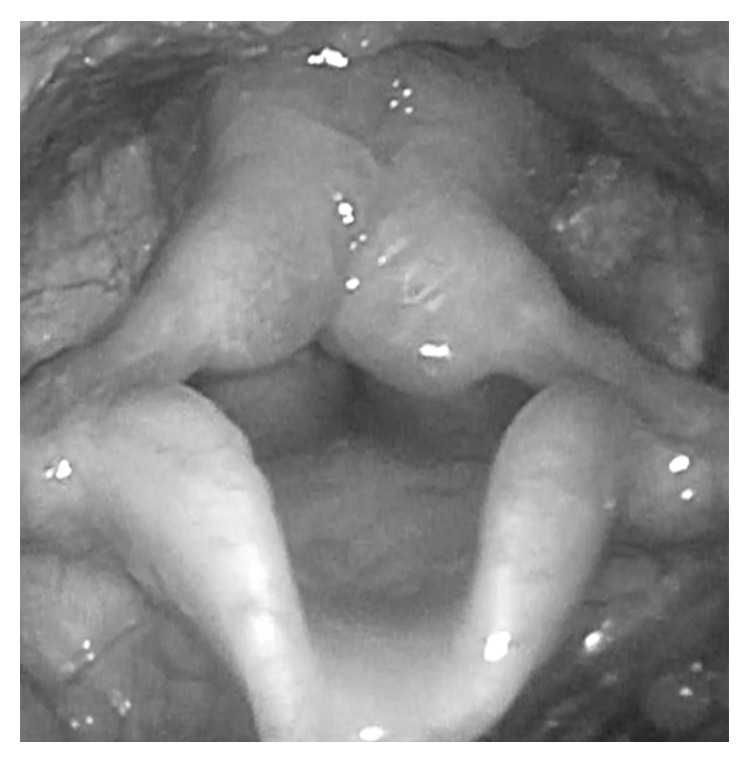
Laryngeal endoscopy revealing swelling in the arytenoid.

**Figure 4:**
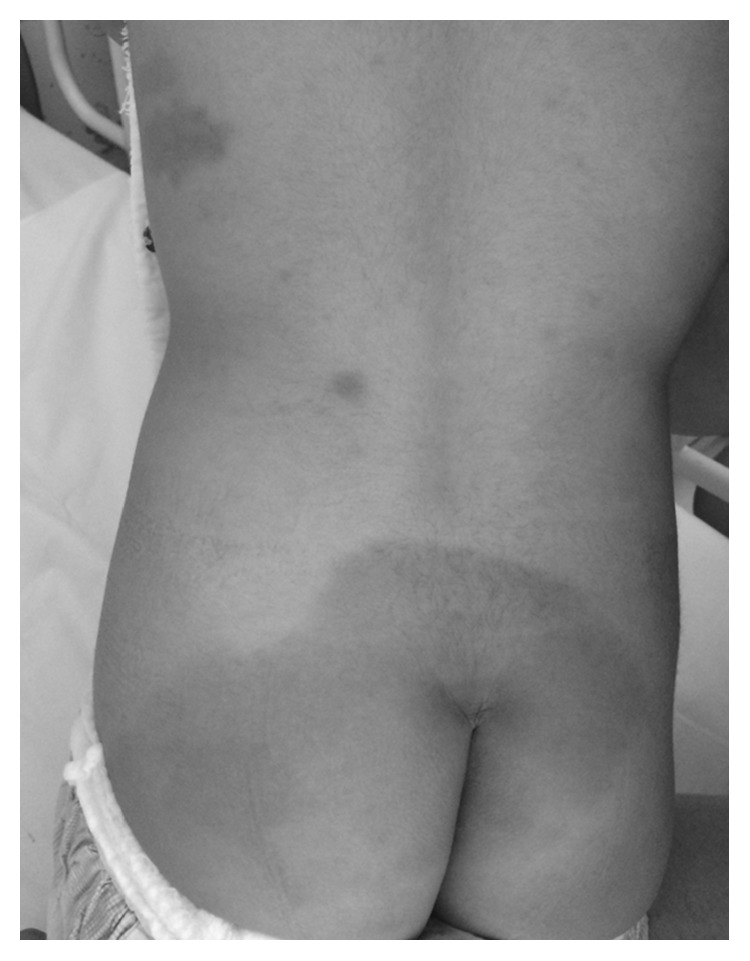
Mongolian spot.

The patient exhibited a coarse face, stiff joints, and claw hand deformity (Figure 1). The result of conditioned orientation response audiometry was 65 dB. Examination of the oropharynx revealed marked hypertrophy of the tonsils, adenoids, and tongue. A lateral neck X-ray revealed obstruction of the nasopharyngeal airway due to adenoid hypertrophy (Figure 2), and the arytenoid was swollen (Figure 3). A sleep study revealed an apnea-hypopnea index of 19.5 events/h, and obstructive sleep apnea was 98.5%. He also exhibited an ectopic Mongolian spot (Figure 4).

**Figure 5:**
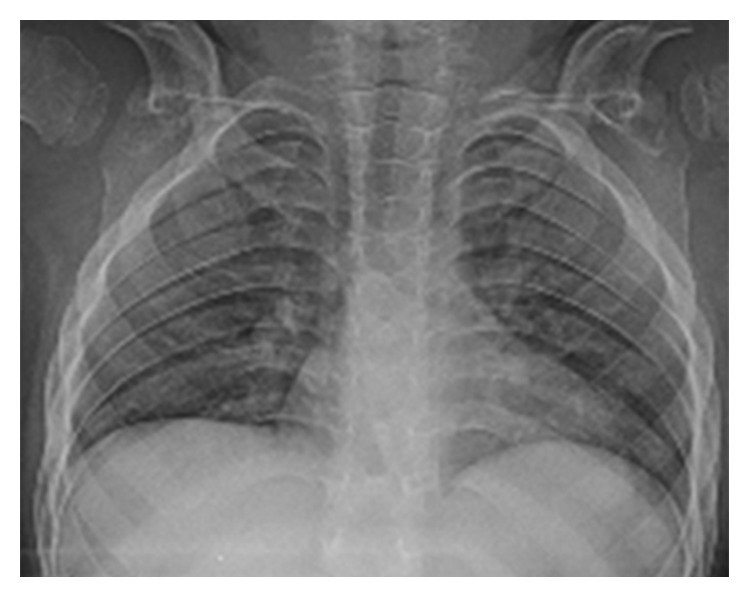
Chest X-ray image revealing oar-like ribs.

**Figure 6:**
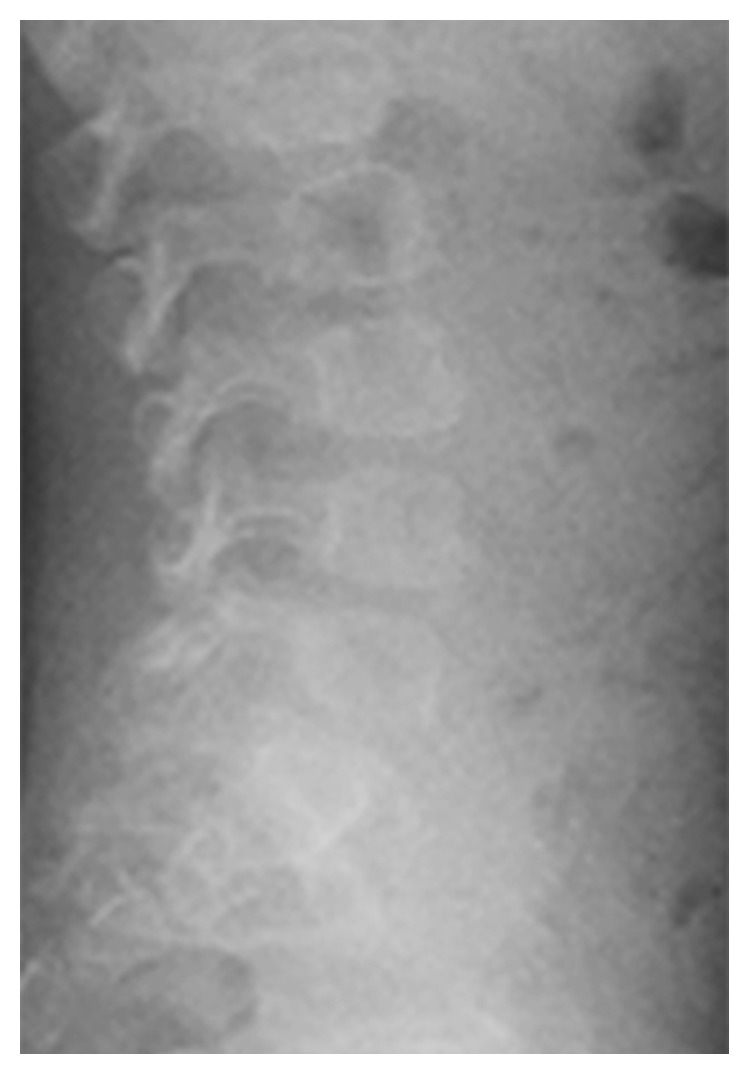
Chest X-ray image revealing egg-shaped vertebra.

**Figure 7:**
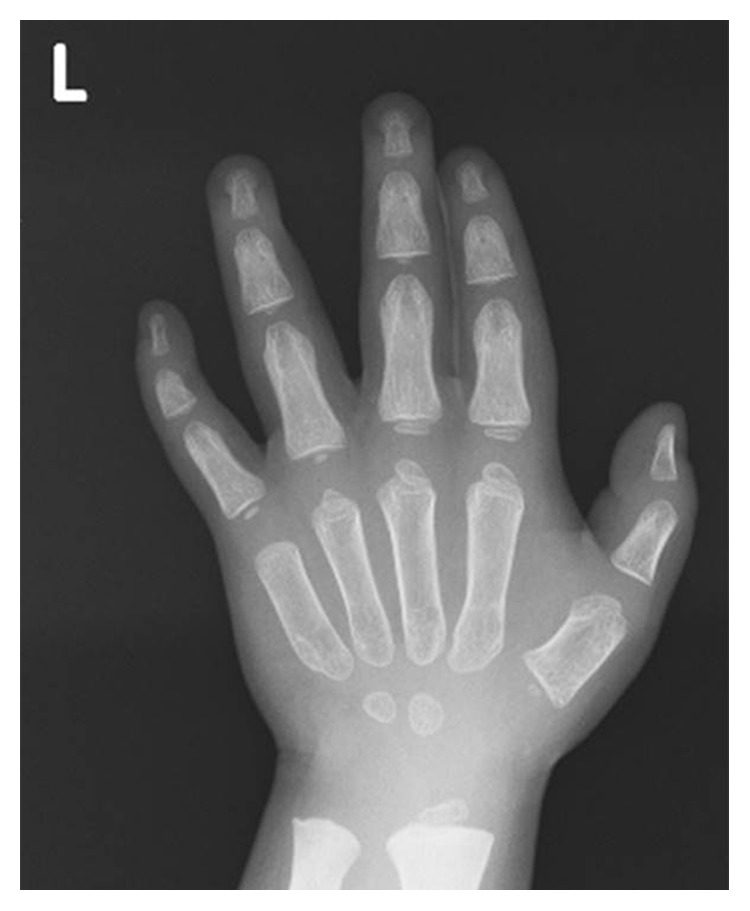
X-ray image of the fingers revealing sharp metacarpals.

**Figure 8:**
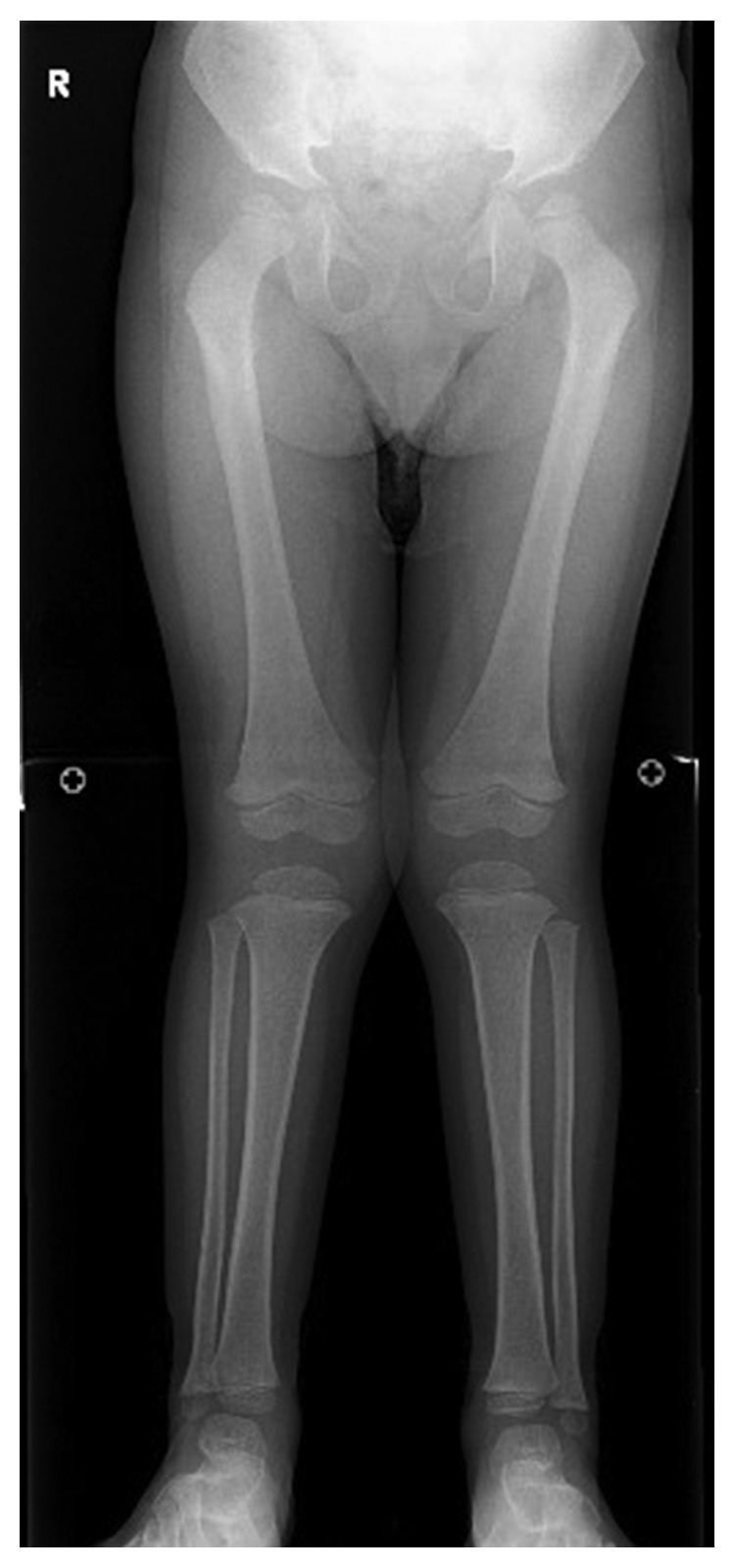
Genu valgum.

**Figure 9:**
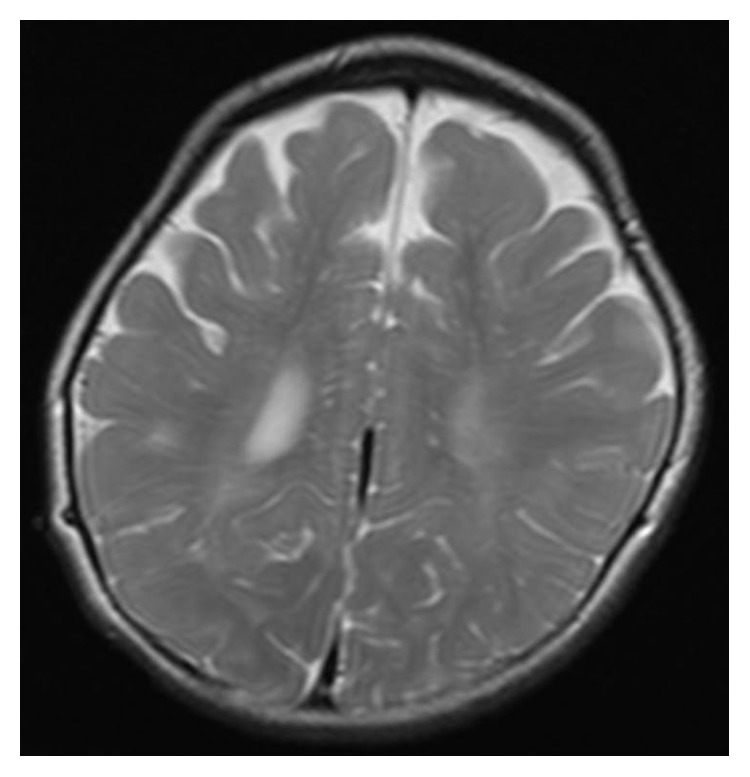
Head magnetic resonance image revealing an enlarged Virchow-Robin space.

The patient underwent adenotonsillectomy. During the operation, intubation was difficult and was performed using a video laryngo scope. The anesthesiologist suggested the possibility of Hunter syndrome. It was difficult to insert the mouth opener and visualize the lower edge of the tonsils during the operation because of tongue hypertrophy. The patient remained intubated in the pediatric intensive care unit for two days after operation because of oropharyngeal swelling, especially the uvula. After two days, he was extubated without problem, and the orthopedics department consulted. Chest radiography revealed oar-like ribs (Figure 5) and an egg-shaped chest vertebra (Figure 6). Sharp metacarpal bones in the fingers (Figure 7) and genu valgum (Figure 8) were also revealed by radiography. Furthermore, head magnetic resonance imaging revealed an enlarged Virchow-Robin space (Figure 9). These findings were reasonably consistent with the characteristics of MPS. Uronic acid testing suggested MPS I or MPS II, and an enzyme activity test for I2S yielded a value of <0.7 nmol/mg protein/4 h. He was diagnosed with Hunter syndrome (MPS II).

Enzyme replacement therapy (ERT) was initiated, which led to improved joint stiffness.

## 3. Discussion

Treatments for MPS include bone marrow transplantation, umbilical cord blood transplantation, ERT, and symptomatic treatment. ERT is not highly effective for the bone and the brain [4]; moreover, once symptoms progress, they are not improved with any therapy [5]. Therefore, early diagnosis and early initiation of treatment is crucial [6]. Important symptoms for early diagnosis are hernia and otitis media [6, 7]. However, these symptoms are common childhood complaints; consequently, it is difficult to definitively associate these symptoms with MPS disorder [3].

We experienced difficult intubation in a case with a medical history of inguinal hernia operation; the patient exhibited an ectopic Mongolian spot, coarse face, stiff joints, and claw hand deformity, some of which are clearly characteristic symptoms of MPS. Cohn et al. reported that these symptoms are a mnemonic screening tool for the diagnosis of Hunter syndrome and, according to that report, our case suggested a likelihood of Hunter syndrome >95% [7]. If otorhinolaryngologists can verify ectopic Mongolian spot(s) and hernia, including hernia operation history, when the refractory otitis media is treated, it may be possible to diagnose MPS earlier, given that many patients with MPS II exhibit some otorhinolaryngological symptoms from younger age [8–10].

Otorhinolaryngologists and pediatricians can suspect MPS, and it is important that both cooperate to diagnose and treat MPS as early as possible.

## 4. Conclusions

Hunter syndrome is a lysosomal disease. We report a case of Hunter syndrome diagnosed by an otorhinolaryngologist. To our knowledge, this is the first reported diagnosis by an otorhinolaryngologist despite the fact otorhinolaryngological symptoms manifest at a young age in this disease. This case report illustrates the significant role of otorhinolaryngologists and the importance of cooperation with pediatricians for early diagnosis and treatment of MPS.
